# Graphene-Based Environmental Sensors: Electrical and Optical Devices

**DOI:** 10.3390/molecules26082165

**Published:** 2021-04-09

**Authors:** Hikari Kitadai, Meng Yuan, Yongqiang Ma, Xi Ling

**Affiliations:** 1 Department of Chemistry, Boston University, Boston, MA 02215, USA; hk91@bu.edu (H.K.); yuanmeng@cau.edu.cn (M.Y.); 2 Department of Applied Chemistry, College of Science, China Agricultural University, Beijing 100193, China; mayongqiang@cau.edu.cn; 3 Division of Materials Science and Engineering, Boston University, Boston, MA 02215, USA; 4 The Photonics Center, Boston University, Boston, MA 02215, USA

**Keywords:** 2D materials, sensing, FETs, spectroscopy, sensitivity, selectivity

## Abstract

In this review paper, we summarized the recent progress of using graphene as a sensing platform for environmental applications. Especially, we highlight the electrical and optical sensing devices developed based on graphene and its derivatives. We discussed the role of graphene in these devices, the sensing mechanisms, and the advantages and disadvantages of specific devices. The approaches to improve the sensitivity and selectivity are also discussed.

## 1. Introduction

Simple and reliable sensors for trace species detection are highly desirable in a spectrum of applications ranging from medical diagnosis, environmental monitoring, industrial and agricultural processes control, and lab-on-a-chip. In the past two decades, various nanomaterials have been explored for sensing applications, including nanoparticles, nanowires, nanotubes, and nanosheets [1,2,3,4,5,6]. Among these nanomaterials, graphene, a two-dimensional (2D) carbon layer, has drawn significant attention in sensing applications due to its novel properties. The unique structure and electronic properties of graphene (e.g., atomically-thin thickness, large specific surface area, high electron mobility, and high sensitivity to electronic perturbations from foreign molecules) are the cornerstones for the development of graphene-based materials as sensing platforms [7,8,9,10,11]. The general working principle of graphene-based sensors rely on detecting perturbations in electrical and optical signals caused by interactions between graphene and a target molecule. To improve these interactions for selective sensing, probes are usually introduced on the surface of a graphene sheet through surface modifications (e.g., chemical linkages) that act as specific binding sites to the target species.

In this review, we highlight the versatility and robustness of graphene as a sensing platform, with emphasis on environmental chemical sensing applications in both liquid and air media. In particular, we focus on two types of sensors: electrical and optical sensors. Electrical sensors based on conductivity changes benefit from graphene’s exceptional carrier transport properties, allowing for improved sensing capabilities. Optical sensors take advantage of graphene’s unique optical properties and show great promise for future applications. The differences between optical and electrical sensors are vast, and it is outside the scope of this review to enumerate them. Instead, we highlight graphene’s versatility and offer practical considerations for sensor designing. In the following, we demonstrate how graphene has been employed in assessing pH and humidity levels, and the presence and concentration of various gaseous and liquid molecules, under different sensing mechanisms.

### 1.1. Graphene: Basic Optical and Electronic Properties

A single layer of graphene consists of sp^2^-hybridized carbon atoms arranged in a honeycomb lattice with a thickness of only 0.34 nm [12,13]. Although both graphene and graphite share the same chemical composition, their properties are distinctively different: whereas single layer graphene is nearly transparent, allowing up to 97.7% of light transmission in the visible range, each successive graphene layer in graphite adds an additional 2.3% opacity yielding its characteristic dark color; [14] graphene is also mechanically flexible and stretchable and, most importantly, while graphite exhibits metallic behavior, graphene is semi-metallic [15].

**Figure 1:**
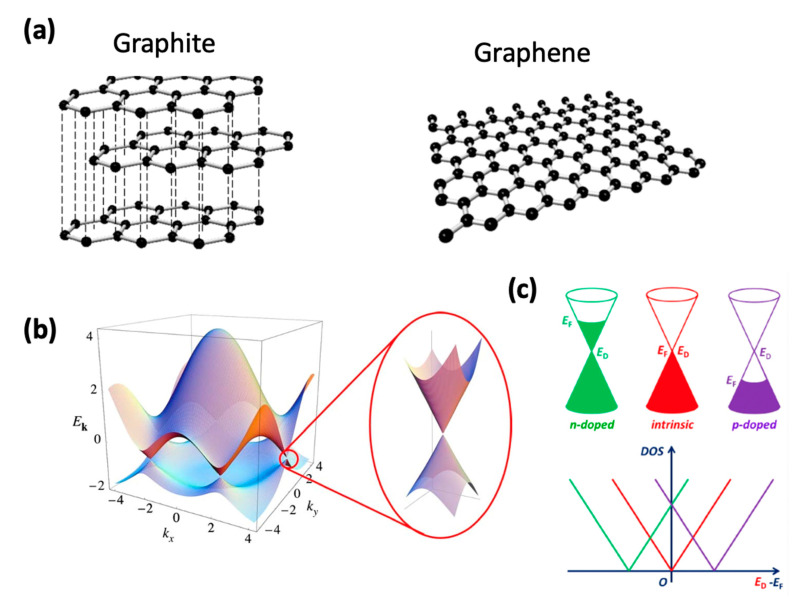
(**a**) Graphite comprised of graphene sheets held together by Van der Waals forces. (**b**) Electronic band structure of graphene showing the Dirac cone, where the conduction band meets the valence band. (**c**) Sketches of the energy-momentum dispersion relation E (k) and the density of states of n-doped graphene (green), intrinsic graphene (red), and p−doped graphene (purple). The doping of graphene leads to a shift of the Fermi level with respect to the Dirac point. Panel (**b**) is reprinted with permission from [15] Copyright 2009, Reviews of Modern Physics.

Graphene’s linear electronic band structure around the K point is largely responsible for the sensitivity to changes in its immediate surroundings. As shown in Figure 1b, at the Dirac point, there is no gap between the valence and the conduction band, meaning the charge carriers have zero rest mass, and electrons and holes are free to move, which gives graphene its semi-metallic nature [15]. Graphene also exhibits a high carrier density (n > 10^12^ cm^−2^), awarding it a superior carrier mobility of over 10^4^ cm^2^/V∙s at room temperature [16,17]. These unique properties render graphene a promising future in sensing applications. Specifically, molecules that approach graphene’s surface induce charge transfers between graphene and the molecules, leading to the doping of graphene positively or negatively [18]. The doping effect changes the relative position between the Dirac point and the Fermi level, which can be reflected in the energy momentum dispersion relation and the density of states, as shown in Figure 1c [19,20]. In intrinsic graphene, the Fermi level is at the Dirac point, while in n-doped graphene, the Fermi level is above the Dirac point; in p-doped graphene, the Fermi level is below the Dirac point. For example, Kim et al. have demonstrated that the work function of graphene oxide can be lowered via wet chemical doping with several alkali metals, including Na, K, Rb, and Cs; subsequent reduction of the oxygen functional groups lowers the work function even further, to as low as 3.02 eV [21]. In a similar study, Miškovíc-Stankoví et al. have shown that Cs doping is also possible during graphene growth, without the need for the wet chemical process, and the work function decreased from 4.5 eV to 3.3 eV [22]. Rhee et al. give an extensive review of the several ways to measure and modulate the working function of graphene [23]. Consequently, assessing changes in the Fermi level allow quantitative measurements of the doping nature of graphene which can be easily done through field effect transistors (FET) devices. Although FET devices are limited to only measuring changes in conductivities, a major advantage of developing onto the FET structure rests on the ease of implementation of such devices when compared to new state-of-the art approaches. New technology is not required for the analysis of the results, and the overall structure is maintained requiring minimal adaptation in the manufacturing process.

### 1.2. Graphene Production

In nature, graphene is found in the form of graphite, where individual layers of graphene are stacked and held together by Van der Waals (VdW) forces (Figure 1a). Graphene was experimentally isolated in 2004 by Geim et al. by cleaving graphite until few layers was achieved [24]. Since then, graphene has been extensively used in research across various fields, ranging from biomedical research to fundamental physics [8,25,26,27,28,29]. The researchers used an adhesive tape to overcome the VdW forces to separate the individual graphene layers, known as the mechanical exfoliation (ME) technique. While this method yields one of the highest graphene grades, as it comes from a single crystal and, therefore, has a lower concentration of defects and impurities, it comes at the expense of relatively small lateral sizes and a long preparation time. The laborious nature of this procedure, together with the low yield, render this technique unsuitable for mass production. Other methods that have shown to be scalable and can be classified into two general categories, one that produces graphene flakes of up to hundreds of micrometers but are discontinuous, and the other produces continuous graphene sheets over a few centimeters but have a higher operating cost.

To mitigate the low yield of ME and scale the exfoliation of bulk graphite into graphene, liquid-mediated exfoliation techniques have been proposed. There are two general types of liquid exfoliation, (1) the intercalation of graphite layers to overcome the VdW forces, and (2) the mechanical exfoliation of graphite into a colloid stabilized by surfactants or solvents. The general approach to graphite intercalation involves oxidizing graphite, to introduce oxygen-containing functional groups, and subsequent sonication. Due to the oxidation step, the resulting graphene oxide flakes need to be reduced to eliminate the oxygen groups. To avoid the oxidation and reduction steps, which can cause permanent structural damage to the sample, a different approach in the technique called the liquid phase exfoliation (LPE) has been under exploration. In the LPE approach bulk graphite is directly sonicated in solvents (e.g., *N*-methyl-2-pyrroidone [30] or dimethylformamide [31]) that have sufficient surface tension for stabilizing the graphene flakes once they are delaminated from the bulk [30,32]. While this procedure is closer to mass production than ME for its scalability, the lateral size distribution is limited to a few micrometers and the number of layers achieved varies from monolayer to multilayers.

Another synthetic route to producing mono- and few-layer graphene with larger lateral sizes (>1 cm^2^) are bottom-up approaches in which individual C atoms are assembled to form graphene. An early method was the controlled sublimation of SiC for the growth of epitaxial graphene. With the desorption of Si, excess carbon on the SiC substrate rearranges in hexagonal lattices. The immediate drawback of this procedure is the high cost of SiC substrates [33]. An alternative method with potentially lower cost is chemical vapor deposition (CVD). In the production of CVD graphene, a hydrocarbon gas (usually methane, CH_4_) is mixed with hydrogen at high temperatures (>1000 °C) in a tube furnace containing a metal substrate with suitable carbon solubility, e.g., Cu or Ni. In the case of low-pressure CVD (LPCVD) the procedure starts by annealing the metal foil under a constant flow of H_2_ in low vacuum, this step reduces the residual oxide layers on the metal surface as well as increases the grain sizes. It is important to achieve higher grain sizes so that the resulting graphene has fewer grain boundaries. After annealing, CH_4_ is introduced to the furnace with a predetermined ratio with the H_2_ gas. Once the reaction is done, the furnace and CH_4_ gas are turned off, and the foil cools down to room temperature still under H_2_ flow. The cooling down step helps avoid excess carbon from aggregating and forming adlayers. The growth step of low solubility substrates (i.e., Cu) occurs when CH_4_ decomposes, and C atoms adsorb on its surface and form a film. In high-solubility substrates (i.e., Ni) the C atoms diffuse into the bulk and graphene growth occurs during the cooling down step when C precipitates back to the surface [34,35,36]. Although the resulting product is polycrystalline, has grain boundaries and adlayers, the overall quality of the graphene may still offset these considerations [37].

## 2. Graphene-Based FET Sensors: Air and Water

**Figure 2:**
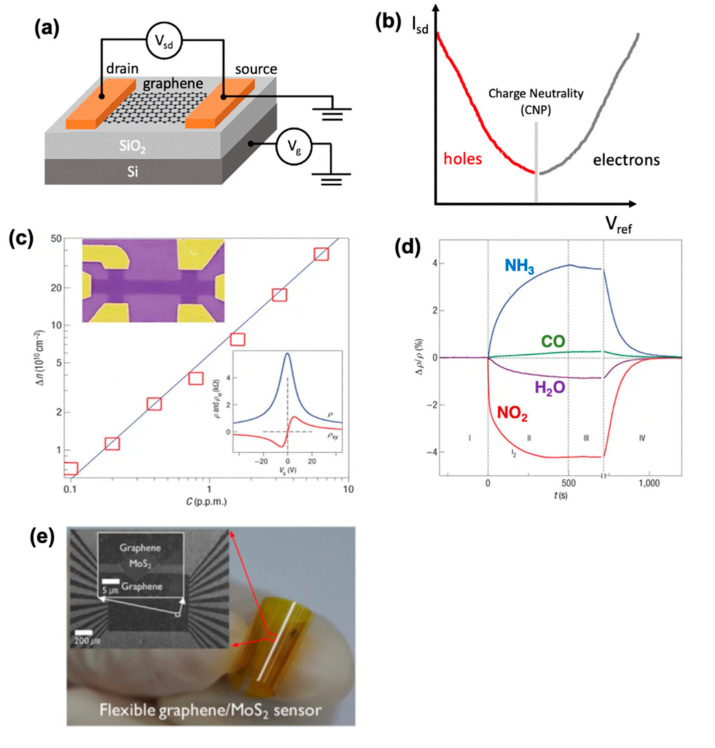
(**a**) Schematic of a back-gated GFET. (**b**) Typical ambipolar transfer characteristics showing that the type of carriers in graphene can continuously be modulated from holes (on the left, in red) to electrons (on the right, in gray) using the field effect. (**c**) The charge carriers in single-layer graphene exposed to different concentrations of NO_2_. Upper inset: Scanning electron micrograph of this device. Lower inset: Characterization of the graphene device by using the electric-field effect. (**d**) Changes in resistivity, Δρ, of graphene by exposure to various gases diluted to 1 ppm. T is response time. Region I: device in vacuum; II: exposure to diluted chemicals; III: evacuation of the experimental setup; and IV: annealing at 150 °C. Inset shows an optical micrograph of the graphene device. I Optical image of flexible graphene/MoS_2_ heterostructured sensor on a bent polyimide substrate. SEM image of the MoS_2_ sensor with patterned graphene electrodes. MoS_2_ flake is bridged by two graphene lines. Panels (**c**,**d**) are reprinted with permission from [39] Copyright 2007, Nature Materials. Panel (**e**) is reprinted with permission from [40] Copyright 2015, American Chemical Society.

Graphene-based electrical sensors are based on FET devices, and work by detecting changes in the conductance of the transducing material (i.e., graphene). Due to the unique linear band structure around the K point, the conductance of the graphene channel is very sensitive to molecular adsorptions on the FET device. Figure 2a shows a typical graphene-based back-gated FET (GFET), which is composed of a source and drain metallic electrodes bridged by a graphene channel and is usually supported by a conductive silicon substrate coated with an insulating dielectric SiO_2_ layer as the back gate. In such devices, the carrier concentration, and thus the conductivity of graphene, can be tuned by the gate voltage. Figure 2b shows a typical measurement where a constant bias voltage (V_sd_) between the graphene channel and the source is applied, and changes in the source-drain current (I_sd_) are recorded. By changing the back-gate voltage (V_g_), the electrochemical potential of the charge carriers (i.e., the Fermi energy) can be modulated. The type of carrier can continuously be tuned from holes (red curve) to electrons (gray curve), with the I_sd_ change following a “V” shape, where the minimum current point marks the transition between p- and n-type, also known as the charge neutrality point (CNP). This behavior is the so-called “ambipolar behavior”.

When exposed to a gaseous environment, gas molecules adsorb on graphene, causing a doping effect to graphene, which, in turn, affects the carrier concentration in graphene. The CNP consequently shifts positively or negatively, depending on whether the gas molecules are p-type or n-type dopant [18]. The degree of the shift can be used to quantify the concentration of gas molecules in the environment. Such devices have been shown useful in assessing gas by-products in manufacturing plants, such as carbon dioxide and ammonia gas—well-known greenhouse gases [38]. Schedin et al. realized the first micrometer-sized graphene sensor and demonstrated its potential by detecting a single gas molecule adsorbed on graphene’s surface (Figure 2c), the highest sensitivity among any detection techniques at the time. To achieve this feat, the authors used a ME graphene, for its inherently low Johnson noise, which led to a high signal-to-noise ratio. The device showed concentration-dependent changes in electrical resistivity when adsorbing NO_2_, H_2_O, NH_3_, and CO gases, allowing for quantitative analysis, shown in Figure 2d. As CO and NH_3_ act as electron donors and NO_2_ and H_2_O act as electron acceptor gases, they were found to strongly adsorb on graphene at room temperature. Moreover, the authors demonstrated the robustness of these devices by recovering them through vacuum annealing at 150 °C suggesting the potential for multiple measurements.

The rise in temperatures around the globe over the past several decades has significantly affected the water cycle. A consequence of these changes is an erratic precipitation pattern which will challenge, among others, the agricultural industry, natural ecosystems, and potable water supplies. Therefore, there is great interest in continuing the development of new technologies in water quality assessment. Following the realization of the GFET gas sensors, liquid-gated GFET sensors were also developed for sensing liquid samples to assess changes in pH, ion concentrations, and contaminants in water samples [38]. The change of I_sd_ versus V_ref_ of the liquid-gated GFETs has similar characteristics to back-gated GFETs, but the gate bias is applied to the liquid medium through a reference electrode (often Ag/AgCl), instead of a dielectric material (e.g., SiO_2_) as in the back-gated configuration.

In the following sections we discuss important considerations for the design of environmental sensors that can affect cost, selectivity, and sensitivity.

### 2.1. GFET Sensors: Graphene Quality Considerations

Despite the high performance of ME graphene samples, they pose a manufacturing challenge for large-scale fabrication as the process is not suitable for automation. CVD graphene, on the other hand, is a scalable fabrication alternative with potential to improve the commercialization of GFET devices. Significant progress in the development of CVD GFET devices has been made over the past decade [41,42,43]. For example, Chen et al. assessed the sensitivity of a CVD GFET sensor when exposed to oxygen (O_2_) at room temperature [44]. The authors found that when O_2_ molecules adsorbed onto the surface of graphene, epoxide and carboxylic groups formed, which significantly decreased the electrical resistivity. The reported limit of detection (LOD) for the O_2_ sensor was about 400 parts per million (ppm).

In a systematic study, Ricciardella et al. investigated how graphene from CVD, ME, and LPE affected the sensitivity of a gas sensing device [45]. The authors exposed the devices to an environment containing NO_2_ in various concentrations, ranging from 0.1 to 1.5 ppm. Not surprisingly, the highest response rate came from the least defective graphene prepared by ME, which was 50% faster than the CVD graphene sensor. While the LPE sensor was also able to detect concentrations down to 0.1 ppm, the response rate was much slower, about a fifth of the ME device. The differences are attributed to the presence of low- and high-energy binding sites stemming from point-defects, generated during graphene preparation. Lower-energy binding sites allow for quicker adsorption, thus, the ME graphene chemosensor shows the highest response rate.

In addition to graphene quality, the effect of the number of graphene layers on the device’s performance has also been studied [28,46]. For example, Li et al. used CVD graphene grown on copper (single layer) and on nickel (few-layer) to detect pH changes in aqueous solutions. In their work, the authors demonstrated that few-layer graphene (FLG) was sensitive to a pH range between 7 and 13, while single layer graphene (SLG) was sensitive to a shorter pH range between 7 and 8 [28]. Additionally, the resolution on the pH scale was one order of magnitude higher in the SLG than that in the FLG devices, 0.01 vs. 0.1 respectively. Although these devices do not cover pH values below 7, they are sensitive from neutral to alkaline environments, and the choice between SLG and FLG will be determined by the application of the device, if the interested pH range lies within physiological processes then SLG is better suited for the task. On the other hand, if the device needs to cover a larger and alkaline pH range then FLG should be chosen.

Considering manufacturing efforts, scalability, and device performance, CVD graphene shows larger commercialization potential in GFET sensors, because it retains the relatively high performance of ME graphene, and has less defects than LPE graphene which hinders carrier mobility.

### 2.2. Functionalization of Graphene for Selectivity Improvement

To improve selectivity, a widely used approach is borrowed from a “lock-and-key” model that preferentially targets the molecule of interest to interact with graphene [47,48]. This is achieved through the addition of functional groups to graphene’s surface [49]. The most commonly used functionalization methods include covalently bonding functional groups (e.g., carboxylic acid, epoxy) on graphene, or adsorbing conjugated organic molecules (e.g., enzyme, sRNA or DNAzyme) through π-π interactions [50,51,52]. Here, we introduce two typical approaches used for graphene functionalization for highly selective GFETs sensing devices.

Covalent functionalization occurs through the formation of new bonds between the sp^2^ backbone of graphene, or graphene oxide, and reactive intermediates, such as carbenes, nitrenes, or free radicals [53]. Generally, graphene oxide is easier to be functionalized through covalent bonding due to dangling oxygenated groups (e.g., carboxylic acid and epoxy) on the surface, compared to the chemically inert surface of intrinsic graphene. However, even though the functionalization can be very stable, it can interfere with the electron transport performance, due to the disruption of graphene’s conjugated π system. Consequently, it is not the most popular approach in the fabrication of GFETs sensors.

Non-covalent functionalization, on the other hand, is a less stable approach to enhance selectivity. To achieve non-covalent functionalization, the devices are usually incubated in the solution of probe molecules (e.g., polymers, enzymes, sRNA or DNAzyme) [54]. This type of functionalization occurs through π-π interactions and does not present considerable changes to the electron transport properties of graphene. Due to its extensive conjugated π system, pristine graphene is an excellent candidate for non-covalent functionalization [54,55]. For example, Zhang et al. used self-assembled 1-octadecanethiol on graphene to sense mercury [56]. In the sensing process, Hg^2+^ interacted strongly with the thiol group in 1-octadecanethiol, and LOD of about 10 mg/L was achieved. Although still not comparable to the guidelines for drinking water set forth by the World Health Organization (WHO) for mercury of 0.006 mg/L [57]. In nature, water samples are often a mixture of several solutes other than a single molecule of interest, such as salts, heavy metals, among others. Therefore, high selectivity to the target species is needed to distinguish from other contaminants in the water. An et al. achieved an improved LOD for Hg^2+^ detection by modifying the surface of graphene with 1,5-diaminonaphthalene (DAN) as a linker through π-π interactions between the phenyl groups on DAN and the π electrons of graphene [58]. Then, the immobilized RNA aptamer was grafted onto graphene, and showed an excellent field-induced response when detecting Hg^2+^. The device showed preferential binding to Hg^2+^ instead of several ions, including Cd^2+^, Co^2+^, Ni^2+^, Na^+^, Pb^2+^, Sr^2+^, Li^+^ and Zn^2+^. The reported LOD for Hg^2+^ was about 6.5 × 10^−6^ mg/L, well below the WHO standard. As many molecules has been explored to “lock” on graphene, [49] more targets can be detected in a highly efficient way with proper “locks” [58].

### 2.3. Graphene Hybrid Structures for Improved Sensitivity

Once selectivity of the target molecule has been achieved, the detection sensitivity should also be improved for sensing of trace-amounts. To this end, several groups have reported that the addition of some metal nanostructures could increase the sensitivity to changes in electron mobility and conductivity [59,60]. Graphene can be doped by the addition of a metallic film or nanoparticle layer, thus modulating the Fermi level: when combined with Al, Ag, or Cu, graphene will be n-doped, and Au or Pt will lead to p-doping of graphene [61]. In addition to modulating the Fermi level, Iqbal et al. demonstrated that the addition of Ag nanoparticles (1.0 M) can significantly increase the electron mobility, from 944 cm^2^ V^−1^ s^−1^ for pristine CVD grown graphene to 1170 cm^2^ V^−1^ s^−1^ with Ag nanoparticles with 200 nm diameter. Further decreasing the nanoparticle diameter to 30 nm leads to an electron mobility of over 1400 cm^2^ V^−1^ s^−1^, almost a 50% increase from pristine graphene [59].

Another route to improve sensitivity that has been under investigation is the vertical stacking of other 2D materials on graphene. For example, Long et al. reported MoS_2_/graphene hybrid aerogel used for NO_2_ detection [62]. The graphene scaffold provided high specific surface area and high electrical and thermal conductivity, and a few-layer MoS_2_ (molybdenum disulfide) sheet could provide a higher sensitivity and selectivity to NO_2_ against H_2_ and CO. The sensor shows an ultralow LOD of 50 ppb NO_2_ at room temperature, much lower than the NO_2_ standard (0.11 ppm) in air quality guidelines of the WHO [63]. The higher selectivity for NO_2_ likely comes as a result of the comparatively larger binding energy than H_2_ and CO when adsorbed on MoS_2_ [64]. An additional benefit of the implementation of other 2D materials is that the GFET device can be made flexible, and has drawn significant interest from both academia [65] and industry [66,67]. Cho et al. fabricated a graphene/MoS_2_ 2D heterostructure based flexible gas sensor, where mechanically exfoliated MoS_2_ flakes were used as the channel material, while the patterned graphene strips were used as electrodes [40]. Figure 2e shows an optical image of the flexible graphene/MoS_2_ heterostructure sensor on a flexible polyimide substrate. The device was able to sense NO_2_ down to 5 ppm, and NH_3_ down to 100 ppm even after 5000 bending cycles. The bending cycles were performed by affixing two opposing sides of the flexible device on two separate arms on an instrument capable of tuning the distance between the arms. The complete bending cycle constituted of two movements, first the arms moved towards each other until the flexible device would bulge to a radius of 1.9 mm, then the arms were allowed to move back to their original positions, making the device flat again.

Higher performance can be achieved by the implementation of both functionalization and graphene-metal hybrids approaches. For example, the WHO Guidelines for drinking-water quality (2011) state that the concentration of Pb^2+^ should not exceed 0.01 ppm [57]. Wen et al. demonstrated their GFET device could detect concentrations several orders of magnitude lower than the WHO standard, about 4 × 10^–6^ ppm [68]. The authors deposited gold nanoparticles (Au NPs) on a graphene channel and modified it with a DNAzyme by non-covalent functionalization. As discussed, such graphene-metal hybrid FETs can reach a low detection limitation comparable to worldwide standards, highlighting the potential for immediate practical applications. In summary, FETs can benefit from the implementation of graphene as the sensing layer for its superior carrier transport properties. Additionally, graphene as a platform is ideal for the introduction of surface modifiers (e.g., chemical linkers, nanoparticles, and 2D hybrid structures) that increase selectivity and sensitivity. Table 1 summarizes GFET chemical sensors discussed here.

## 3. Graphene-Based Optical Sensors

The versatility of graphene is also showcased in optical sensors, which enjoy a plethora of sensing mechanisms that can provide more information about the target species than field-effect-transistors. In essence, optical sensors are instruments capable of measuring the interaction between electromagnetic radiation and matter; here, we review how spectroscopies can benefit from graphene. A major advantage of optical sensors arises from the fact that they are capable of probing energetic states (e.g., electronic, vibrational, rotational) which are intrinsic to the target species, paving way for directly detecting the presence of the target analyte. In stark contrast to GFET devices that, instead, rely on indirect measurements (i.e., conductivity changes in the graphene-molecule complex) to detect the target analyte.

There are numerous optical sensors with distinct sensing mechanisms, and while all are not presented here, we highlight the use of graphene in a variety of spectroscopic modalities ranging from colorimetric sensors, that require little sample preparation and almost no post analysis processing, to more complex techniques like Raman spectroscopy that provide specific spectral signatures but require specialized instrumentation and knowledge to operate. We begin by reviewing sensors that detect changes in the optical properties of the graphene-analyte complex.

### 3.1. Colorimetric Sensors

One of the simplest optical sensor designs comes in the form of colorimetric sensors, because of its streamlined operation, both in data acquisition and analysis. This type of sensor consists of a substrate to hold the graphene, and the graphene film itself. To operate the sensor, the user only needs to expose the device to the sensing medium (i.e., air, water) for a color change to occur in real time. Although they are easy to operate, the underlying mechanisms for the color change can vary significantly, and here we highlight two distinct examples of how colorimetric sensors can take advantage of graphene. The first one relies on an optical inference caused by the swelling of the graphene film that ultimately affects the reflection of visible light, in other words, the reflectance changes. The second method involves grafting molecules on the surface of graphene that emit light in the presence of the target species, a process called fluorescence resonance energy transfer (FRET).

**Figure 3:**
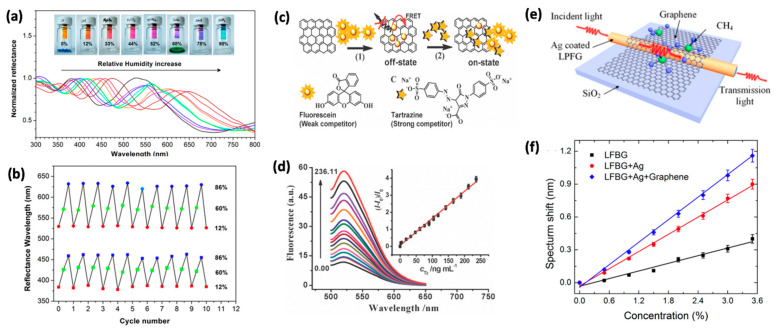
(**a**) Color change of GOs film when exposure to various relative humidity and UV–VIS reflecting spectral shifts of a GOs film under different humidity conditions at 25 °C. (**b**) Reversible conversion from the dual-colorimetric of GO multilayers by alternately exposing to selected relative humidity. (**c**) Chemical structures of fluorescein and tartrazine. Assay principle illustrates a turn-off/on fluorescence response of rGO–dye reporter pairs in the course of a displacement transformation. (**d**) Fluorescence emission spectra of the fluorescent sensor in the presence of different concentrations of tartrazine. (**e**) Schematic of the graphene-based LPFG SPR sensor. (**f**) Resonance wavelength shift of SPR sensor versus concentration of methane. Panels (**a**,**b**) are reprinted with permission from [87] Copyright 2015, American Chemical Society. Panels (**c**,**d**) are reprinted with permission from [91] Copyright 2012, Royal Society of Chemistry. Panels (**e**,**f**) are reprinted from [92] open access.

2D graphene, especially, graphene oxide, has demonstrated exceptional promise for humidity sensing, because of their super permeability that arises from the rich oxygen-containing functional groups on the surface [86]. The functional groups of graphene oxide (i.e., hydrophilic hydroxyl, epoxy, and carboxylic groups) were tuned for monitoring humidity. Chi et al. reported a colorimetric sensor for monitoring relative humidities up to 98% (Figure 3a) [87]. A thin film of GO was coated on a SiO_2_ substrate and exposed to environments with different humidity levels. To realize different levels of humidity, the researchers prepared several vials with varying water vapor pressures from saturated salt solutions. The relative humidities were 12% (LiCl), 33% (MgCl_2_), 44% (K_2_CO_3_), 52% (Mg(NO_3_)_2_), 68% (CuCl_2_), 75% (NaCl), and 98% (PbNO_3_). The thin film changed colors from light blue to orange, according to their relative humidity levels (Figure 3b). The device allowed naked-eye visualization of humidity levels within a fast speed of 250 ms. The humidity-dependent color changes of the GO film are attributed to the swelling of the GO layers when exposed to moisture, because of its superpermeability to water molecules. An increase in relative humidity leads to more nanopores in the GO film that allow for the insertion of more water molecules which in turn swell the film. The perceived color changes come as a result of an optical interference between the reflected light of the upper and lower interfaces of the device (air/GO film and GO film/SiO_2_ substrate, respectively) as the GO film thickness changes. More recently, Gong et al. proposed in 2018 a similar colorimetric device capable of detecting ammonium, methanol, and ethanol gases [88]. The sensor was especially sensitive to ethanol gas, with an LOD of 3.3 ppm and a fast response time of 120 ms. Additionally, because this type of sensors relies on the adsorption of gas molecules on the surface of graphene, desorption can also be induced, and the same device can be reused multiple times. The authors estimate it takes 80 ms for desorption to occur, needing only to expose the device to the ambient air atmosphere, before taking the next measurement. The recoverability of the device was demonstrated by monitoring the average reflectance spectrum peak positions upon adsorption and desorption of EtOH, 577 nm and 527 nm, respectively. The adsorption/desorption cycle was repeated 20 times with 40 s interval between them, and all peak shifts were within 5 nm of their respective positions. As these reflectance-based colorimetric sensors don’t require electrical power to function, they are an attractive option for quick field measurements.

Although it is desirable to fabricate sensors with the least amount of complexity (for economic or durability concerns), their technical simplicity may pose limitations on their practical use. It is not possible for graphene, or its derivatives, to detect all possible target analytes by themselves and the addition of functional groups as anchor points may be required—much like the approach described in Section 2.2. We review a similar approach that instead of anchoring the target molecule to a binding site on the surface, functional groups in the form of “light-switches” are added to the surface of graphene that are tailored to turn-on under certain environmental conditions [89]. For example, in 2014, Kim et al. demonstrated a photoluminescent pH sensor that when dispersed in water could detect a pH range from 1 to 7 [90]. To realize this feat, the authors made clever use of graphene’s fluorescence quenching capabilities, by grafting two differently colored fluorophores on graphene that are sensitive to either acidic or neutral pH. As both chromophores make contact with graphene, their fluorescence is quenched and no light is emitted. However, when the sensor is exposed to acidic (neutral) media, one of the fluorophores will emit an orange (blue) hue light. In another example, Huang et al. introduced a sensitive, rapid, label-free fluorescent method using reduced graphene oxide (rGO) to identify tartrazine (Figure 3c,d) [91]. The authors first bound a fluorescein to rGO, resulting in the quenching of the fluorescence. As tartrazine was added into the system, it competed with fluorescein to bind with rGO, leading to the desorption of some fluorescein molecules from rGO. Thus, fluorescence recovery was observed afterwards. By quantifying the fluorescence recovery using fluorescence spectroscopy, the concentration of tartrazine was determined based on the linear relationship between the fluorescence quenching intensity and the concentration of the tartrazine.

### 3.2. Optical Fiber-Based Sensors

Optical fibers offer a great way to guide light over long distances, due to their incredibly high total internal reflection that allows for minimal propagation loss inside the cladding. Over the last few decades, demands for miniaturization of sensors for real-time and remote monitoring has positioned optical fibers as a pivotal platform for its small size, flexibility, chemical inertness and, more importantly, insusceptibility to external electromagnetic interference [93,94].

However, the same cladding that protects the fiber from interference and signal loss also prevents analytes from interacting with the light in the core of the fiber [95]. To overcome this challenge several approaches have been proposed, such as polishing [96], chemical etching [97], and tapering [95] to name a few, and they all share a similar trait: exposure of the core to the elements. With the removal of the cladding, light propagating through the fiber is able to interact with its surrounding. The sensing mechanism of these optical fibers rests in exciting the evanescent field so that it can interact with its surrounding medium. However, because the core of the fiber is usually made of primarily silica and is, therefore, inert, there is little selectivity towards any given analyte, and a sensing layer is required to draw the molecules close to the exposed portion of the fiber [98,99,100]. In the following, we present two types of optical fiber sensors, one is based on reflectance changes, like the colorimetric sensor discussed above, and another sensor that is based on a phenomenon called surface plasmon resonance.

Rosli et al. prepared a tapered optical fiber sensor to detect aqueous ethanol by measuring differences in the reflectance spectra. The fiber tips were coated with rGO by drop casting and acted as the sensing layer for ethanol. The reflectance response of the rGO coated fiber tip reduced linearly, upon exposure to ethanol concentrations ranging between 20–80% [101]. Zhang et al. reported a polymer optical fiber with graphene film deposited on the distal end of the optical fiber as an acetone sensor [102]. When the acetone vapor molecules were adsorbed on the graphene film, the change of the reflectance on the spectra showed a two-fold improved sensitivity than without it. Detection of acetone vapor concentrations as low as 44 ppm was achieved on such graphene modified sensor, while original sensor can only detect acetone down to about 70 ppm.

Another sensing technique that has enjoyed significant progress from the development of optical fibers are Surface Plasmon Resonance (SPR) sensors [103,104,105]. SPR sensors are based on an optical phenomenon in which the collective coherent oscillation of free electrons, usually of a metal, is induced by an incoming electromagnetic field at the interface between a metal and a dielectric [106]. These charge density oscillations are called surface plasmon polaritons (SPP). The SPPs form an electric field that propagates outward into the surrounding medium, namely the evanescent field, which is sensitive to changes in the refractive index. SPR sensors, then, are sensors capable of measuring refractive-index changes at the sensing surface.

Recently, the addition of graphene to Au and Ag systems have shown to increase the SPR signal change when compared to the bare metal [107,108]. Zhu et al. coated monolayer graphene onto the silver film surface of long-period fiber grating (LPFG) and used the SPR property of such structure to sense methane gas, Figure 3e [92]. At a 3.6% methane concentration, a three-fold improved sensitivity in spectral shift was observed compared with the traditional LPFG sensor, from 0.4 to 1.2 nm, and 1.3 times better than the Ag-coated LPFG SPR sensor, with a spectral shift of 0.9 nm, (Figure 3f). It was attributed to a graphene-induced increased intensity of the evanescent field on the surface of the fiber and, thus, the interaction between SPR wave and the target molecules was enhanced [92,109].

### 3.3. Graphene-Mediated Surface Enhanced Raman Spectroscopy

Raman spectroscopy is a fingerprinting technique that is non-contact and non-destructive. An advantage of Raman spectroscopy is that it can assess the intrinsic vibrational signature of the target molecule and the signal is not inferred from changes in the system. However, a major drawback to spontaneous Raman scattering is that it is a relatively weak process: around one in a million photons will undergo Raman scattering. The remaining photons will scatter photons with the same energy as the incident light, also called Rayleigh scattering (after Lord Rayleigh, who discovered this phenomenon). To mitigate the low efficiency, many efforts in far-field, near-field, and non-linear Raman spectroscopy have been made to increase the scattered signal, notable mentions include resonance Raman, surface enhanced Raman, and stimulated Raman spectroscopy.

In practical terms, surface enhanced Raman spectroscopy (SERS) offers a balanced solution between signal intensity and relatively low sample preparation. In SERS the experimental setup is identical to traditional Raman spectrometers, the only difference lies in the sample preparation: the molecules must be in close proximity to the SERS substrate. Surface enhanced Raman spectroscopy works primarily through two mechanisms, by (1) increasing the probability that a given mode will interact with the incoming photons, or (2) intensifying the incoming or scattered photons. In reality, the SERS signal is usually a combination of both processes.

**Figure 4:**
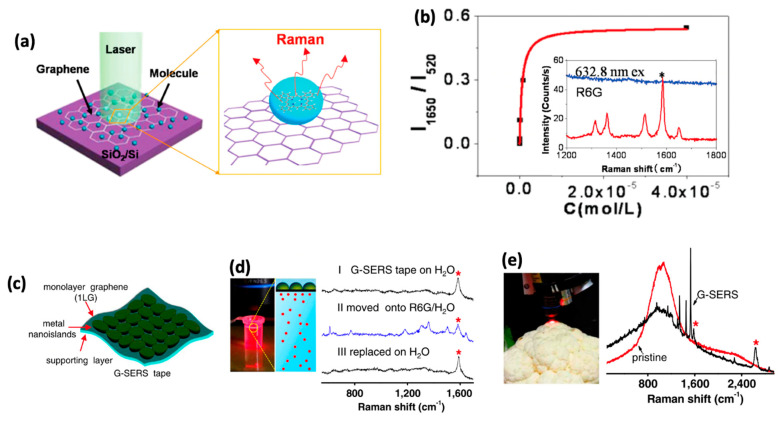
(**a**) Schematic representation of graphene enhanced Raman spectroscopy. (**b**) Raman intensity vs. concentration for R6G dye; inset shows representative graphene enhanced Raman spectrum of R6G (red) compared to the molecules adsorbed on SiO_2_/Si. (**c**) Sketch of G-SERS tape with labeled components. (**d**) Real-time and reversible characterization of an R6G aqueous solution (1 × 10^−5^ M). (**e**) Pristine (red) and G-SERS (black) Raman spectra of a cauliflower submerged in a 1 × 10^−5^ M R6G solution. * marks the enhanced G-band. Panels (**a**,**b**) are reprinted with permission from [110] Copyright 2010, American Chemistry Society. Panels (**c**–**e**) are reprinted with permission from [111] Copyright 2012, National Academy of Sciences.

By the end of 2009, Ling et al. made the first observation of a surface-enhanced Raman scattering (SERS) effect on graphene. The authors demonstrated that dye molecules (possible waterways contaminants) deposited on graphene gave significantly higher Raman signals than those deposited on the bare substrates (Figure 4a) [110]. The researchers compared the Raman intensities of certain dyes (i.e., rhodamine 6g, crystal violet, phthalocyanine) deposited onto bare SiO_2_/Si substrates with those deposited on ME graphene. Following this discovery, the authors termed the phenomenon graphene-enhanced Raman spectroscopy, or GERS for short.

The advantages of using graphene as a platform for Raman enhancement were readily appreciated as the authors found that no conspicuous signals were observed for R6G with a 632.8 nm excitation wavelength on the bare SiO_2_/Si, however with the addition of graphene those signals were clearly observed (inset of Figure 4b). A concentration dependence study revealed that graphene was able to detect R6G down to 8 × 10^−10^ M, a remarkable finding considering no extra steps in sample preparation were required to achieve such a low detection limit (Figure 4b). Although the precise mechanism behind the GERS effect is still under debate, the leading understanding attributes the enhancement to an increase in Raman scattering cross section, as the intensification of incoming and scattered photons is unlikely because graphene does not support surface plasmons under visible light excitation. A major factor contributing to the Raman enhancement on graphene is the coupling between graphene’s fermi level and the molecule’s highest occupied molecular orbital and lowest unoccupied molecular orbital.

While graphene can enhance Raman signals, there are many other factors that make it a prime candidate for environmental sensing. In addition to enhancing Raman signals, graphene can also take advantage of its large surface area to volume ratio for the addition of functional groups allowing for specific binding to target molecules, thus increasing both selectivity and sensitivity. By combining traditional SERS substrates (i.e., noble metals) and graphene, both the Raman scattering cross section and the incident and scattering photons get enhanced, together making a graphene-mediated surface-enhanced Raman scattering substrate, or G-SERS.

Such designs are under current exploration with ever more sensitive, selective, and efficient SERS substrates. Xu et al. have proposed a G-SERS tape consisting of a polymer layer supporting a graphene/metal hybrid structure, the design of the tape takes advantage of the fact that graphene offers an ideal surface for molecules to adsorb on, and gold nanoparticles to enhance the incoming and scattered photons (Figure 4c) [111]. The resulting structure is a sensitive, flexible, and reusable proof-of-concept device that can aid future research to develop tailored solutions to specific sensing applications. To demonstrate its recyclability, the researchers took Raman spectra of the G-SERS tape onto a water solution containing R6G molecules (1 × 10^−5^ M) and compared it to the tape placed only on water without the dye (Figure 4d). When the tape is placed on water, no R6G modes are visible in Spectrum I in Figure 4d; once the tape is moved to the R6G solution, clear peaks are observed in Spectrum II in Figure 4d. The molecules can be washed off simply by placing the tape in water, evidenced by the lack of conspicuous R6G Raman peaks in spectrum III in Figure 4d.

The G-SERS tape is also remarkably versatile, as it can also be used with both liquid and solid samples. To demonstrate that analysis of trace amounts of analytes with the G-SERS tape works on any surface with arbitrary morphology, the authors submerged a cauliflower head into a copper phthalocyanine (CuPc) solution (1 × 10^−5^ M) and took a Raman spectrum which showed no discernible CuPc peaks. However, when placing the G-SERS tape under the laser spot on the cauliflower clear CuPc peaks are observed, Figure 4e.

## 4. Conclusions

In this review, we have highlighted the recent progress in the development of graphene-based electrical and optical sensors for environmental applications, for example, in assessing pH and humidity levels, and the presence and concentration of various molecules. Graphene-based FET sensors can be made to detect target molecules in both gas and liquid samples, and due to their similarity with FETs in operation, their incorporation in existing sensing systems is viable. While graphene-based optical sensors require more intricate setups (e.g., light source and detector), they offer the advantage of being non-contact and are promising for multiplexing in the case of optical-fiber sensors and G-SERS.

There are several aspects that make graphene an ideal sensing platform. A major one is graphene’s unique linear band structure around the K point that makes it sensitive to perturbations caused by adsorbed molecules; here we reviewed how these changes can be probed electrically and optically. The atomically thin nature of graphene allows the possible miniaturization of devices, so that more sensors could be packed in the same area. Additionally, we also detailed general steps towards performance improvements in terms of selectivity and sensitivity through the addition of surface modifiers that (1) preferentially bind to the target molecule and (2) increase graphene’s sensitivity to external perturbations, respectively. Further research on surface modifiers is likely to solidify graphene as a sensing platform.

The future of graphene sensors is promising but some challenges need to be overcome. While there has been significant effort on realizing proof-of-concept devices, the transition from laboratory to market primarily hinges on the performance of these sensors. Although high selectivity and sensitivity can be achieved in laboratory settings, it is not clear how these devices will fare in real-world scenarios. It is rare to have contaminant-free samples when dealing with environmental applications and the effect of contamination might hinder sensor readings. Although we have reviewed that graphene-based sensors do not always need to come single crystal graphene samples and lower grades are usually sufficient, the biggest hurdle for widespread adoption of this new technology still rests on the availability of graphene, both in terms of quality and cost. We also anticipate the integration of wireless technology to graphene sensors will significantly expand the adoption of these devices in the age of the Internet-of-Things for their real-time measurement capabilities.

## Figures and Tables

**Table 1:** Reported GFET sensors for environmental detection.

Analyte	Surface Modifier	Graphene Type	Temperature (°C)	Sensitivity	Response Time	Concentration	Safety Guidelines	Ref.
pH	N/A	Graphene/CVD	25	pH 0.01	3.11–3.7	pH 7–13	N/A	[28]
Hg^2+^	1-octadecanethiol	Graphene/ME	N/A	N/A	N/A	10 ppm	0.006 ppm ^b^	[56]
Pb^2+^	DNAzyme	Graphene/ME	N/A	N/A	2 min	37.5 ^a^–23,800 ng/L	0.01 ppm ^b^	[69]
Pb^2+^	AuNP—Glutathione	rGO/Hummers	N/A	N/A	N/A	163.7 ^a^–500 ng/L		[70]
NH_3_	N/A	Graphene/CVD	RT	N/A	1 min	20–100 ppm	300 ppm ^c^	[71]
NH_3_	NO_2_-doped	Graphene/CVD	RT	N/A	50 min (100 ppm)	200 ppb ^a^		[72]
NH_3_	N/A	Graphene/CVD	NA	N/A	33 s	130 ppb ^a^ 9–2400 ppm		[73]
H_2_	SO_2_ nanoparticles	Graphene/ME	50	N/A	~1 s	1 ^a^–100 ppm	N/A	[74]
H_2_	PtNPs	GO	RT	N/A	30 s	200–500 ppm		[75]
SO_2_	N/A	Graphene/CVD	40–100	N/A	2.5 min	50 ppm	0.17 ppm ^b^	[76]
SO_2_	TiO_2_	rGO	RT	N/A	~120 s	1 ^a^–5000 ppb		[77]
H_2_S	Cu_2_O nanocrystals	Graphene sheet	RT	11%	N/A	5 ^a^–100 ppb	10–20 ppm ^d^	[78]
NO_2_	SiC	Graphene single layer/ epitaxial	RT	2.5–50 ppm	N/A	2.5 ppm ^a^	0.11 ppm ^b^	[79]
NO_2_	N/A	Graphene (Stretchable devices)/CVD	N/A	NA	1 min	200 ppm		[80]
NO_2_	Mogul-patterned substrate	rGO	RT	2.5–25 ppm	1 min	2.5 ppm		[81]
Orthophosphate	Ferritin	rGO/Hummers	RT	N/A	Few seconds	26 nM ^a^	N/A	[82]
Antibiotic	PASE—DNAcapturestrands	Graphene/CVD	RT	0.001 × 10^9^ M^−1^	200 s	11.5 × 10^−^^9^ M ^a^	N/A	[83]

^a^ LOD; RT room temperature; ^b^ [63]; ^c^ [84]; ^d^ [85].

## References

[1] Abdel-Karim R., Reda Y., Abdel-Fattah A. (2020). Review—Nanostructured Materials-Based Nanosensors. J. Electrochem. Soc..

[2] Vikesland P.J. (2018). Nanosensors for water quality monitoring. Nat. Nanotechnol..

[3] Li M., Gou H., Al-Ogaidi I., Wu N. (2013). Nanostructured sensors for detection of heavy metals: A review. ACS Sustain. Chem. Eng..

[4] Liu Y., Deng Y., Dong H., Liu K., He N. (2017). Progress on sensors based on nanomaterials for rapid detection of heavy metal ions. Sci. China Chem..

[5] Willner M.R., Vikesland P.J. (2018). Nanomaterial enabled sensors for environmental contaminants. J. Nanobiotechnol..

[6] Khin M.M., Nair A.S., Babu V.J., Murugan R., Ramakrishna S. (2012). A review on nanomaterials for environmental remediation. Energy Environ. Sci..

[7] Chang J., Zhou G., Christensen E.R., Heideman R., Chen J. (2014). Graphene-based sensors for detection of heavy metals in water: A review Chemosensors and Chemoreception. Anal. Bioanal. Chem..

[8] Coroş M., Pruneanu S., Stefan-van Staden R.-I. (2020). Review—Recent Progress in the Graphene-Based Electrochemical Sensors and Biosensors. J. Electrochem. Soc..

[9] Justino C.I.L., Gomes A.R., Freitas A.C., Duarte A.C., Rocha-Santos T.A.P. (2017). Graphene based sensors and biosensors. TrAC Trends Anal. Chem..

[10] Nag A., Mitra A., Mukhopadhyay S.C. (2018). Graphene and its sensor-based applications: A review. Sensors Actuators, A Phys..

[11] Yao Y., Ping J. (2018). Recent advances in graphene-based freestanding paper-like materials for sensing applications. TrAC Trends Anal. Chem..

[12] Lee H., Park J.Y. (2019). Height determination of single-layer graphene on mica at controlled humidity using atomic force microscopy. Rev. Sci. Instrum..

[13] Lu J.P. (1997). Elastic Properties of Carbon Nanotubes and Nanoropes. Phys. Rev. Lett..

[14] Nair R.R., Blake P., Grigorenko A.N., Novoselov K.S., Booth T.J., Stauber T., Peres N.M.R., Geim A.K. (2008). Fine structure constant defines visual transparency of graphene. Science.

[15] Castro Neto A.H., Guinea F., Peres N.M.R., Novoselov K.S., Geim A.K. (2009). The electronic properties of graphene. Rev. Mod. Phys..

[16] Hwang E.H., Adam S., Sarma S. (2007). Das Carrier transport in two-dimensional graphene layers. Phys. Rev. Lett..

[17] Yin Y., Cheng Z., Wang L., Jin K., Wang W. (2014). Graphene, a material for high temperature devices—Intrinsic carrier density, carrier drift velocity, and lattice energy. Sci. Rep..

[18] Fu W., Jiang L., van Geest E.P., Lima L.M.C., Schneider G.F. (2017). Sensing at the Surface of Graphene Field-Effect Transistors. Adv. Mater..

[19] Matković A., Kratzer M., Kaufmann B., Vujin J., Gajić R., Teichert C. (2017). Probing charge transfer between molecular semiconductors and graphene. Sci. Rep..

[20] Gao L. (2014). Probing Electronic Properties of Graphene on the Atomic Scale by Scanning Tunneling Microscopy and Spectroscopy. Graphene 2D Mater..

[21] Naghdi S., Song H.Y., Várez A., Rhee K.Y., Kim S.W. (2020). Engineering the electrical and optical properties of graphene oxide via simultaneous alkali metal doping and thermal annealing. J. Mater. Res. Technol..

[22] Naghdi S., Nešovic K., Sánchez-Arriaga G., Song H.Y., Kim S.W., Rhee K.Y., Miškovic-Stankovic V. (2020). The effect of cesium dopant on APCVD graphene coating on copper. J. Mater. Res. Technol..

[23] Naghdi S., Sanchez-Arriaga G., Rhee K.Y. (2019). Tuning the work function of graphene toward application as anode and cathode. J. Alloys Compd..

[24] Novoselov K.S., Geim A.K., Morozov S.V., Jiang D., Zhang Y., Dubonos S.V., Grigorieva I.V., Firsov A.A. (2004). Electric Field Effect in Atomically Thin Carbon Films. Science.

[25] Li Y., Feng Z., Huang L., Essa K., Bilotti E., Zhang H., Peijs T., Hao L. (2019). Additive manufacturing high performance graphene-based composites: A review. Compos. Part A Appl. Sci. Manuf..

[26] Zubiarrain-Laserna A., Kruse P. (2020). Review—Graphene-Based Water Quality Sensors. J. Electrochem. Soc..

[27] Samaddar P., Son Y.S., Tsang D.C.W., Kim K.H., Kumar S. (2018). Progress in graphene-based materials as superior media for sensing, sorption, and separation of gaseous pollutants. Coord. Chem. Rev..

[28] Li X., Shi J., Pang J., Liu W., Liu H., Wang X. (2014). Graphene channel liquid container field effect transistor as ph sensor. J. Nanomater..

[29] Huang H., Su S., Wu N., Wan H., Wan S., Bi H., Sun L. (2019). Graphene-based sensors for human health monitoring. Front. Chem..

[30] Hernandez Y., Nicolosi V., Lotya M., Blighe F.M., Sun Z., De S., McGovern I.T., Holland B., Byrne M., Gun’Ko Y.K. (2008). High-yield production of graphene by liquid-phase exfoliation of graphite. Nat. Nanotechnol..

[31] Hernandez Y., Lotya M., Rickard D., Bergin S.D., Coleman J.N. (2010). Measurement of multicomponent solubility parameters for graphene facilitates solvent discovery. Langmuir.

[32] Li Z., Young R.J., Backes C., Zhao W., Zhang X., Zhukov A.A., Tillotson E., Conlan A.P., Ding F., Haigh S.J. (2020). Mechanisms of Liquid-Phase Exfoliation for the Production of Graphene. ACS Nano.

[33] De Heer W.A., Berger C., Ruan M., Sprinkle M., Li X., Hu Y., Zhang B., Hankinson J., Conrad E. (2011). Large area and structured epitaxial graphene produced by confinement controlled sublimation of silicon carbide. Proc. Natl. Acad. Sci. USA.

[34] Dayou S., Vigolo B., Ghanbaja J., Medjahdi G., Ahmad Thirmizir M.Z., Pauzi H., Mohamed A.R. (2017). Direct Chemical Vapor Deposition Growth of Graphene Nanosheets on Supported Copper Oxide. Catal. Letters.

[35] Wang X., You H., Liu F., Li M., Wan L., Li S., Li Q., Xu Y., Tian R., Yu Z. (2009). Large-scale synthesis of few-layered graphene using CVD. Chem. Vap. Depos..

[36] Shan C., Tang H., Wong T., He L., Lee S.T. (2012). Facile synthesis of a large quantity of graphene by chemical vapor deposition: An advanced catalyst carrier. Adv. Mater..

[37] Brownson D.A.C., Varey S.A., Hussain F., Haigh S.J., Banks C.E. (2014). Electrochemical properties of CVD grown pristine graphene: Monolayer- vs. quasi-graphene. Nanoscale.

[38] Singh E., Meyyappan M., Nalwa H.S. (2017). Flexible Graphene-Based Wearable Gas and Chemical Sensors. ACS Appl. Mater. Interfaces.

[39] Schedin F., Geim A.K., Morozov S.V., Hill E.W., Blake P., Katsnelson M.I., Novoselov K.S. (2007). Detection of individual gas molecules adsorbed on graphene. Nat. Mater..

[40] Cho B., Yoon J., Lim S.K., Kim A.R., Kim D.-H., Park S.-G., Kwon J.-D., Lee Y.-J., Lee K.-H., Lee B.H. (2015). Chemical Sensing of 2D Graphene/MoS _2_ Heterostructure device. ACS Appl. Mater. Interfaces.

[41] Liao L., Duan X. (2012). Graphene for radio frequency electronics. Mater. Today.

[42] Petrone N., Meric I., Chari T., Shepard K.L., Hone J. (2015). Graphene field-effect transistors for radio-frequency flexible electronics. IEEE J. Electron Devices Soc..

[43] Fisichella G., Lo Verso S., Di Marco S., Vinciguerra V., Schilirò E., Di Franco S., Lo Nigro R., Roccaforte F., Zurutuza A., Centeno A. (2017). Advances in the fabrication of graphene transistors on flexible substrates. Beilstein J. Nanotechnol..

[44] Chen C.W., Hung S.C., Yang M.D., Yeh C.W., Wu C.H., Chi G.C., Ren F., Pearton S.J. (2011). Oxygen sensors made by monolayer graphene under room temperature. Appl. Phys. Lett..

[45] Ricciardella F., Vollebregt S., Polichetti T., Miscuglio M., Alfano B., Miglietta M.L., Massera E., Di Francia G., Sarro P.M. (2017). Effects of graphene defects on gas sensing properties towards NO2 detection. Nanoscale.

[46] Goh M.S., Pumera M. (2011). Graphene-based electrochemical sensor for detection of 2,4,6- trinitrotoluene (TNT) in seawater: The comparison of single-, few-, and multilayer graphene nanoribbons and graphite microparticles. Anal. Bioanal. Chem..

[47] Campos R., Borme J., Guerreiro J.R., Machado G., Cerqueira M.F., Petrovykh D.Y., Alpuim P. (2019). Attomolar label-free detection of dna hybridization with electrolyte-gated graphene field-effect transistors. ACS Sensors.

[48] Ohno Y., Maehashi K., Matsumoto K. (2010). Label-free biosensors based on aptamer-modified graphene field-effect transistors. J. Am. Chem. Soc..

[49] Kemp K.C., Georgakilas V., Otyepka M., Bourlinos A.B., Chandra V., Kim N., Kemp K.C., Hobza P., Zboril R., Kim K.S. (2012). Functionalization of Graphene: Covalent and Non- Covalent Approaches, Derivatives and Applications Functionalization of Graphene: Covalent and Non-Covalent Approaches, Derivatives and Applications. Chem. Rev..

[50] Alshammari A., Posner M.G., Upadhyay A., Marken F., Bagby S., Ilie A. (2016). A Modular Bioplatform Based on a Versatile Supramolecular Multienzyme Complex Directly Attached to Graphene. ACS Appl. Mater. Interfaces.

[51] Gao J., Gao Y., Han Y., Pang J., Wang C., Wang Y., Liu H., Zhang Y., Han L. (2020). Ultrasensitive Label-free MiRNA Sensing Based on a Flexible Graphene Field-Effect Transistor without Functionalization. ACS Appl. Electron. Mater..

[52] Cai B., Wang S., Huang L., Ning Y., Zhang Z., Zhang G.J. (2014). Ultrasensitive label-free detection of PNA-DNA hybridization by reduced graphene oxide field-effect transistor biosensor. ACS Nano.

[53] Criado A., Melchionna M., Marchesan S., Prato M. (2015). The Covalent Functionalization of Graphene on Substrates. Angew. Chemie Int. Ed..

[54] Georgakilas V., Tiwari J.N., Kemp K.C., Perman J.A., Bourlinos A.B., Kim K.S., Zboril R. (2016). Noncovalent Functionalization of Graphene and Graphene Oxide for Energy Materials, Biosensing, Catalytic, and Biomedical Applications. Chem. Rev..

[55] Jang A.R., Jeon E.K., Kang D., Kim G., Kim B.S., Kang D.J., Shin H.S. (2012). Reversibly light-modulated Dirac point of graphene functionalized with spiropyran. ACS Nano.

[56] Zhang T., Cheng Z., Wang Y., Li Z., Wang C., Li Y., Fang Y. (2010). Self-assembled 1-octadecanethiol monolayers on graphene for mercury detection. Nano Lett..

[57] World Health Organization (2011). Guidelines for Drinking-Water Quality.

[58] An J.H., Park S.J., Kwon O.S., Bae J., Jang J. (2013). High-Performance Flexible Graphene Aptasensor for Mercury Detection in Mussels. ACS Nano.

[59] Iqbal M.W., Shahzad K., Ateeq H., Aslam I., Aftab S., Azam S., Kamran M.A., Khan M.F. (2020). An effectual enhancement to the electrical conductivity of graphene FET by silver nanoparticles. Diam. Relat. Mater..

[60] Oh J., Lee J.S., Jun J., Kim S.G., Jang J. (2017). Ultrasensitive and Selective Organic FET-type Nonenzymatic Dopamine Sensor Based on Platinum Nanoparticles-Decorated Reduced Graphene Oxide. ACS Appl. Mater. Interfaces.

[61] Khomyakov P.A., Giovannetti G., Rusu P.C., Brocks G., Van Den Brink J., Kelly P.J. (2009). First-principles study of the interaction and charge transfer between graphene and metals. Phys. Rev. B Condens. Matter Mater. Phys..

[62] Long H., Harley-Trochimczyk A., Pham T., Tang Z., Shi T., Zettl A., Carraro C., Worsley M.A., Maboudian R. (2016). High Surface Area MoS2/Graphene Hybrid Aerogel for Ultrasensitive NO2 Detection. Adv. Funct. Mater..

[63] World Health Organization (2010). WHO Guidelines for Indoor Air Quality: Selected Pollutants.

[64] Yue Q., Shao Z., Chang S., Li J. (2013). Adsorption of gas molecules on monolayer MoS_2_ and effect of applied electric field. Nanoscale Res. Lett..

[65] Carey T., Cacovich S., Divitini G., Ren J., Mansouri A., Kim J.M., Wang C., Ducati C., Sordan R., Torrisi F. (2017). Fully inkjet-printed two-dimensional material field-effect heterojunctions for wearable and textile electronics. Nat. Commun..

[66] Kim H., Kim Y., Kim K.S., Jeong H.Y., Jang A.R., Han S.H., Yoon D.H., Suh K.S., Shin H.S., Kim T. (2013). Flexible thermochromic window based on hybridized VO2/graphene. ACS Nano.

[67] De Fazio D., Goykhman I., Yoon D., Bruna M., Eiden A., Milana S., Sassi U., Barbone M., Dumcenco D., Marinov K. (2016). High Responsivity, Large-Area Graphene/MoS2 Flexible Photodetectors. ACS Nano.

[68] Wen Y., Li F.Y., Dong X., Zhang J., Xiong Q., Chen P. (2013). The Electrical Detection of Lead Ions Using Gold-Nanoparticle- and DNAzyme-Functionalized Graphene Device. Adv. Healthc. Mater..

[69] Wang C., Cui X., Li Y., Li H., Huang L., Bi J., Luo J., Ma L.Q., Zhou W., Cao Y. (2016). A label-free and portable graphene FET aptasensor for children blood lead detection. Sci. Rep..

[70] Zhou G., Chang J., Cui S., Pu H., Wen Z., Chen J. (2014). Real-time, selective detection of Pb^2+^ in water using a reduced graphene oxide/gold nanoparticle field-effect transistor device. ACS Appl. Mater. Interfaces.

[71] Ben Aziza Z., Zhang Q., Baillargeat D. (2014). Graphene/mica based ammonia gas sensors. Appl. Phys. Lett..

[72] Mortazavi Zanjani S.M., Sadeghi M.M., Holt M., Chowdhury S.F., Tao L., Akinwande D. (2016). Enhanced sensitivity of graphene ammonia gas sensors using molecular doping. Appl. Phys. Lett..

[73] Inaba A., Yoo K., Takei Y., Matsumoto K., Shimoyama I. (2014). Ammonia gas sensing using a graphene field-effect transistor gated by ionic liquid. Sensors Actuators, B Chem..

[74] Zhang Z., Zou X., Xu L., Liao L., Liu W., Ho J., Xiao X., Jiang C., Li J. (2015). Hydrogen gas sensor based on metal oxide nanoparticles decorated graphene transistor. Nanoscale.

[75] Wang J., Rathi S., Singh B., Lee I., Joh H.I., Kim G.H. (2015). Alternating Current Dielectrophoresis Optimization of Pt-Decorated Graphene Oxide Nanostructures for Proficient Hydrogen Gas Sensor. ACS Appl. Mater. Interfaces.

[76] Ren Y., Zhu C., Cai W., Li H., Ji H., Kholmanov I., Wu Y., Piner R.D., Ruoff R.S. (2012). Detection of sulfur dioxide gas with graphene field effect transistor. Appl. Phys. Lett..

[77] Zhang D., Liu J., Jiang C., Li P., Sun Y. (2017). High-performance sulfur dioxide sensing properties of layer-by-layer self-assembled titania-modified graphene hybrid nanocomposite. Sens. Actuators B Chem..

[78] Zhou L., Shen F., Tian X., Wang D., Zhang T., Chen W. (2013). Stable Cu_2_O nanocrystals grown on functionalized graphene sheets and room temperature H2S gas sensing with ultrahigh sensitivity. Nanoscale.

[79] Pearce R., Iakimov T., Andersson M., Hultman L., Spetz A.L., Yakimova R. (2011). Epitaxially grown graphene based gas sensors for ultra sensitive NO 2 detection. Sens. Actuators B Chem..

[80] Yun J., Lim Y., Jang G.N., Kim D., Lee S.J., Park H., Hong S.Y., Lee G., Zi G., Ha J.S. (2016). Stretchable patterned graphene gas sensor driven by integrated micro-supercapacitor array. Nano Energy.

[81] Lee H.B., Bae C.W., Duy L.T., Sohn I.Y., Kim D.I., Song Y.J., Kim Y.J., Lee N.E. (2016). Mogul-Patterned Elastomeric Substrate for Stretchable Electronics. Adv. Mater..

[82] Mao S., Pu H., Chang J., Sui X., Zhou G., Ren R., Chen Y., Chen J. (2017). Ultrasensitive detection of orthophosphate ions with reduced graphene oxide/ferritin field-effect transistor sensors. Environ. Sci. Nano.

[83] Wang C., Li Y., Zhu Y., Zhou X., Lin Q., He M. (2016). High-κ Solid-Gate Transistor Configured Graphene Biosensor with Fully Integrated Structure and Enhanced Sensitivity. Adv. Funct. Mater..

[84] NIOSH (1994). Documentation for Immediately Dangerous to Life or Health Concentrations.

[85] World Health Organization, WHO Regional Office for Europe (2000). Air Quality Guidelines for Europe.

[86] Nair R.R., Wu H.A., Jayaram P.N., Grigorieva I.V., Geim A.K. (2012). Unimpeded Permeation of Water Through Helium-Leak–Tight Graphene-Based Membranes. Science.

[87] Chi H., Liu Y.J., Wang F., He C. (2015). Highly sensitive and fast response colorimetric humidity sensors based on graphene oxides film. ACS Appl. Mater. Interfaces.

[88] Gong T., Zhang X., Fu Y., Zhou G., Chi H., Li T. (2018). A facile fabrication of colorimetric graphene oxide reflecting films for ultrasensitive optical gas sensing. Sensors Actuators B Chem..

[89] Chen G., Song F., Xiong X., Peng X. (2013). Fluorescent nanosensors based on fluorescence resonance energy transfer (FRET). Ind. Eng. Chem. Res..

[90] Paek K., Yang H., Lee J., Park J., Kim B.J. (2014). Efficient colorimetric pH sensor based on responsive polymer-quantum dot integrated graphene oxide. ACS Nano.

[91] Huang S.T., Shi Y., Li N.B., Luo H.Q. (2012). Sensitive turn-on fluorescent detection of tartrazine based on fluorescence resonance energy transfer. Chem. Commun..

[92] Wei W., Nong J., Zhang G., Tang L., Jiang X., Chen N., Luo S., Lan G., Zhu Y. (2017). Graphene-based long-period fiber grating surface plasmon resonance sensor for high-sensitivity gas sensing. Sensors.

[93] Yin M., Gu B., Zhao Q., Qian J., Zhang A., An Q., He S. (2011). Highly sensitive and fast responsive fiber-optic modal interferometric pH sensor based on polyelectrolyte complex and polyelectrolyte self-assembled nanocoating. Anal. Bioanal. Chem..

[94] Chocarro-Ruiz B., Fernández-Gavela A., Herranz S., Lechuga L.M. (2017). Nanophotonic label-free biosensors for environmental monitoring. Curr. Opin. Biotechnol..

[95] Leung A., Shankar P.M., Mutharasan R. (2007). A review of fiber-optic biosensors. Sensors Actuators B Chem..

[96] Chandani S.M., Jaeger N.A.F. (2005). Fiber-optic temperature sensor using evanescent fields in D fibers. IEEE Photonics Technol. Lett..

[97] Veselov A.A., Abraham B.G., Lemmetyinen H., Karp M.T., Tkachenko N.V. (2012). Photochemical properties and sensor applications of modified yellow fluorescent protein (YFP) covalently attached to the surfaces of etched optical fibers (EOFs). Anal. Bioanal. Chem..

[98] Elosua C., Arregui F.J., Del Villar I., Ruiz-Zamarreño C., Corres J.M., Bariain C., Goicoechea J., Hernaez M., Rivero P.J., Socorro A.B. (2017). Micro and nanostructured materials for the development of optical fibre sensors. Sensors.

[99] McDonagh C., Burke C.S., MacCraith B.D. (2008). Optical Chemical Sensors. Chem. Rev..

[100] Cubillas A.M., Unterkofler S., Euser T.G., Etzold B.J.M., Jones A.C., Sadler P.J., Wasserscheid P., Russell P.S.J. (2013). Photonic crystal fibres for chemical sensing and photochemistry. Chem. Soc. Rev..

[101] Rosli M.A.A., Arasu P.T., Noor A.S.M., Lim H.N., Huang N.M. (2016). Reduced Graphene Oxide nano-composites layer on fiber optic tip sensor reflectance response for sensing of aqueous ethanol. J. Eur. Opt. Soc..

[102] Zhang H., Kulkarni A., Kim H., Woo D., Kim Y.-J., Hong B.H., Choi J.-B., Kim T. (2011). Detection of acetone vapor using graphene on polymer optical fiber. J. Nanosci. Nanotechnol..

[103] Yang Z., Chen S., Fang P., Ren B., Girault H.H., Tian Z. (2013). LSPR properties of metal nanoparticles adsorbed at a liquid-liquid interface. Phys. Chem. Chem. Phys..

[104] Law W.-C., Yong K.-T., Baev A., Hu R., Prasad P.N. (2009). Nanoparticle enhanced surface plasmon resonance biosensing: Application of gold nanorods. Opt. Express.

[105] Verma R., Gupta B.D., Jha R. (2011). Sensitivity enhancement of a surface plasmon resonance based biomolecules sensor using graphene and silicon layers. Sensors Actuators B Chem..

[106] Raether H. (1988). Surface plasmons on smooth surfaces. Surface Plasmons on Smooth and Rough Surfaces and on Gratings.

[107] Choi S.H., Kim Y.L., Byun K.M. (2011). Graphene-on-silver substrates for sensitive surface plasmon resonance imaging biosensors. Opt. Express.

[108] Alharbi R., Irannejad M., Yavuz M. (2019). A short review on the role of the metal-graphene hybrid nanostructure in promoting the localized surface plasmon resonance sensor performance. Sensors.

[109] Reed J.C., Zhu H., Zhu A.Y., Li C., Cubukcu E. (2012). Graphene-enabled silver nanoantenna sensors. Nano Lett..

[110] Ling X., Xie L., Fang Y., Xu H., Zhang H., Kong J., Dresselhaus M.S., Zhang J., Liu Z. (2010). Can graphene be used as a substrate for Raman enhancement?. Nano Lett..

[111] Xu W., Ling X., Xiao J., Dresselhaus M.S., Kong J., Xu H., Liu Z., Zhang J. (2012). Surface enhanced Raman spectroscopy on a flat graphene surface. Proc. Natl. Acad. Sci. USA.

